# Fecal Myeloperoxidase Levels Reflect Disease Activity in Children With Crohn’s Disease

**DOI:** 10.1093/ibd/izae262

**Published:** 2024-11-11

**Authors:** Teagan S Edwards, Shaun S C Ho, Stephanie C Brown, Laura Appleton, Briana R Smith, Grace M Borichevsky, Akhilesh Swaminathan, Christopher M A Frampton, Richard B Gearry, Anthony J Kettle, Andrew S Day

**Affiliations:** Department of Paediatrics, University of Otago, Christchurch, New Zealand; Department of Paediatrics, University of Otago, Christchurch, New Zealand; Department of Gastroenterology and Clinical Nutrition, The Royal Children’s Hospital Melbourne, Melbourne, Victoria, Australia; Department of Paediatrics, University of Melbourne, Melbourne, Victoria, Australia; Murdoch Children’s Research Institute, Melbourne, Victoria, Australia; Department of Paediatrics, University of Otago, Christchurch, New Zealand; Department of Paediatrics, University of Otago, Christchurch, New Zealand; Department of Pathology and Biomedical Sciences, Mātai Hāora—Centre for Redox Biology and Medicine, University of Otago, Christchurch, New Zealand; Department of Pathology and Biomedical Sciences, Mātai Hāora—Centre for Redox Biology and Medicine, University of Otago, Christchurch, New Zealand; Department of Medicine, University of Otago, Christchurch, New Zealand; Department of Medicine, University of Otago, Christchurch, New Zealand; Department of Medicine, University of Otago, Christchurch, New Zealand; Department of Gastroenterology, Christchurch Hospital, Christchurch, New Zealand; Department of Pathology and Biomedical Sciences, Mātai Hāora—Centre for Redox Biology and Medicine, University of Otago, Christchurch, New Zealand; Department of Paediatrics, University of Otago, Christchurch, New Zealand

**Keywords:** biomarkers, myeloperoxidase, pediatrics

## Abstract

**Background:**

Crohn’s disease (CD) is a major form of inflammatory bowel disease (IBD) which has relapsing and remitting symptoms. Better ways to detect and monitor active disease are required for early diagnosis and optimal outcomes. We assessed fecal myeloperoxidase (fMPO), a neutrophil-derived enzyme that produces hypochlorous acid, as a marker of disease activity in children with CD.

**Methods:**

This observational study assessed myeloperoxidase (MPO) levels in fecal samples from children aged <17 years with CD (51 with active or 42 inactive disease) measured by enzyme-linked immunosorbent assay (ELISA) and compared to controls (35 healthy siblings and 15 unrelated well children). Results were correlated with fecal calprotectin, serum C-reactive protein, urinary glutathione sulfonamide (a biomarker of hypochlorous acid), and disease activity scores. Differences between groups were assessed by analysis of variance. Receiver-operating-characteristic curves were used to assess how biomarkers predicted disease and disease activity.

**Results:**

Fecal myeloperoxidase activity and fMPO protein correlated with fecal calprotectin (*r* = 0.78, *P* < .0001, and *r* = 0.81, *P* < .0001, respectively). Fecal myeloperoxidase activity and protein levels were significantly higher (*P* ≤ .0001) in individuals with active disease compared to healthy sibling controls, unrelated well children, and those with inactive disease. A 9.7 µg/g fMPO protein cutoff distinguished inactive from active disease (sensitivity = 75%, specificity = 76%). Urinary GSA was elevated in children with active disease (*P* < .0001) and correlated with fMPO protein (*r* = 0.43, *P* = .0002) in a subset of 72 children with IBD and controls.

**Conclusions:**

Fecal myeloperoxidase may be superior to fCal at reflecting disease severity in children with CD and produces the damaging oxidant hypochlorous acid during active inflammation.

## Graphical Abstract

**Graphical Abstract:**
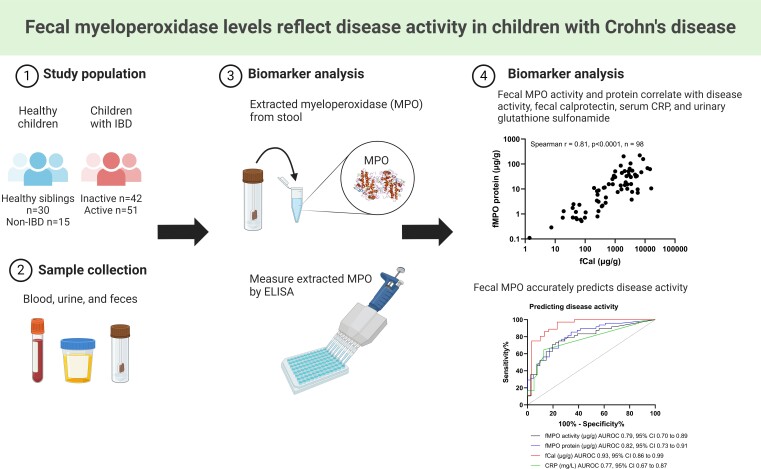

**Key Messages**

**What is already known?**

Noninvasive methods to diagnose or monitor inflammatory bowel disease are required to enable early diagnosis and optimize outcomes.

**What is new here?**

This study identified that fecal myeloperoxidase (fMPO) can predict the presence of Crohn’s disease (CD) and disease activity in children, performing similarly to routinely used fecal calprotectin.

**How can this study help patient care?**

Measurement of fMPO, particularly activity, is a fast and inexpensive test that could aid in the diagnosis of CD and disease monitoring in children, this test could be developed into a point-of-care test for use at home or in the clinic.

## Introduction

Inflammatory bowel disease (IBD), encompassing Crohn’s disease (CD) and ulcerative colitis (UC), is becoming increasingly common in children worldwide.^1^ Inflammatory bowel disease is an incurable condition characterized by relapsing and remitting gut symptoms that can include diarrhea, abdominal pain, and fatigue. Up to a quarter of patients are diagnosed in childhood, with delayed diagnosis leading to worse outcomes such as impaired growth, nutrition, and psychological well-being. Various treatments are available to reduce inflammation and maintain remission; none are curative. Better ways to detect and monitor active disease are required to enable early diagnosis and to optimize care and outcomes for children with CD.

Current methods for diagnosis and monitoring this condition, including colonoscopy with biopsy sampling, are invasive and costly. However, researchers have yet to identify a noninvasive test that can replace these methods. Several stool-based markers have been evaluated as indicators of gut inflammation. Fecal calprotectin (fCal) measurement is widely accepted as a generally reliable test for prediagnostic screening and disease monitoring.^2^ However, it is expensive and can take days to obtain results. Furthermore, detection of fCal may be less reliable in some situations, including in younger children,^3^ and when calprotectin is oxidized,^4^ with oxidation occurring at sites of infection and inflammation resulting in degradation and release of specific peptides.^4,5^

Myeloperoxidase (MPO), an abundant neutrophil granule enzyme, shows promise as a biomarker. It plays a critical role in the innate immune response by killing invading pathogens through its production of the potent oxidant hypochlorous acid.^6^ Myeloperoxidase-derived hypochlorous acid also reacts with a wide range of biological molecules such as proteins, carbohydrates, lipids, and nucleic acids.^7^ In addition, excessive or misplaced activation of neutrophils damages host tissues. Myeloperoxidase-derived oxidants have been shown to contribute to the pathogenesis of various inflammatory diseases including IBD,^8^ cystic fibrosis (CF),^9^ rheumatoid arthritis,^10^ cardiovascular disease,^11^ and chronic obstructive pulmonary disease.^12^

Despite a broad understanding of the roles that MPO plays in inflammation, limited studies have assessed MPO as a biomarker in IBD. Previous studies have measured fecal myeloperoxidase (fMPO) levels in small numbers of adults with active IBD (mainly with UC).^13-16^ However, fMPO extraction and measurement and the indices used to assess IBD activity were variable. Recent work demonstrated that fMPO is an accurate biomarker of endoscopic inflammation in adults with IBD, with elevated levels predicting a more complicated disease course.^17,18^ Serum MPO levels have also been shown to be elevated in children with IBD (*n* = 35) compared to controls (*n* = 32).^19^ However, to the best of our knowledge, there have been no studies assessing fMPO as a marker of inflammation in children with CD.

Myeloperoxidase-derived hypochlorous acid oxidizes glutathione to produce mainly glutathione disulfide (GSSG) but it also produces the specific product glutathione sulfonamide (GSA).^20^ Unlike oxidized glutathione (GSSG), GSA is not a substrate of glutathione reductase and is a stable marker of neutrophil oxidant activity. Previous studies have demonstrated that urinary GSA may be an effective noninvasive marker of neutrophilic inflammation in early CF lung disease.^9^ The identification of urinary GSA in children with CD would indicate that MPO is active and generating hypochlorous acid during inflammation.

This was an exploratory study to ascertain whether fMPO is superior to routinely measured fCal and can reflect disease severity in children with CD. Secondary goals assessed whether GSA could be detected in the urine to determine whether MPO is more active in producing hypochlorous acid during inflammation in children with CD compared to healthy children.

## Materials and Methods

### Patient Recruitment

Children aged <17 years of age residing in the Canterbury region of New Zealand presenting with clinical suspicion of IBD, known IBD, or control children without IBD (healthy siblings and unrelated well children) were recruited between September 2012 and July 2020. Children provided informed assent and parents/legal guardians provided written informed consent. Exclusion criteria included other pre-existing chronic inflammatory diseases (such as eosinophilic esophagitis or autoimmune disease), any underlying renal diseases, prior gastrointestinal surgery (except appendicectomy), or use of nonsteroidal anti-inflammatory medications within the week prior to enrollment.

Clinical suspicion of IBD was defined as the presence of intestinal or extraintestinal symptoms suggestive of IBD. Children with clinical suspicion of IBD underwent standard clinical assessments including routine blood and stool tests, imaging, and endoscopy (gastroscopy and ileocolonoscopy with histology of mucosal biopsies). The final diagnosis was made by the treating physician with classification as CD or UC based upon the revised Porto criteria.^21^ Inflammatory bowel disease unclassified (IBD-U) was used when the diagnosis of IBD was clear but unable to distinguish between CD or UC. Patients diagnosed with IBD-U were determined by their treating physician to either have disease favoring CD or UC and they were grouped accordingly. For the purposes of this study, only samples from children with IBD diagnosed with CD or IBD-U favoring CD were analyzed.

The study participants were categorized into 4 groups: active CD, inactive CD, non-IBD, and healthy sibling controls. The active CD group included children with newly diagnosed untreated CD, or those with known disease and a current relapse requiring reinduction treatment. Patients with relapsed disease were excluded if they had received any reinduction therapy (such as corticosteroids) within the 4 weeks prior to enrollment. The inactive CD group included children with known CD who were clinically in remission based on standard disease activity scores^22^ and had stable CD treatment in the three months prior to enrollment. The non-IBD group included participants who underwent investigations for gastrointestinal symptoms and from whom IBD was excluded. The healthy sibling group consisted of siblings of children with known IBD who were reported by their parents/carers to be healthy.

### Sample Collection and Storage

Recruited participants consented to the collection of a venous blood sample at the time of diagnostic colonoscopy prior to the administration of anesthetic medications. Blood samples were centrifuged at 4 °C and plasma was collected. Collected plasma was aliquoted and frozen at −80 °C for future analysis. Plasma samples were not used as part of this study. Participants also provided baseline urine and stool samples where possible. Stool and urine samples were stored in insulated bags with freezer packs overnight at 4 °C before being delivered to a local laboratory and aliquoted and frozen at −80 °C for subsequent analysis. Basic demographics, medications, medical history, CRP results, endoscopic imaging, histologic findings, and disease activity scores were collected and recorded. Study data were collected and managed using Research Electronic Data Capture (REDCap) electronic data capture tools hosted by the University of Otago.^23^

### Assessment of Disease Activity

Disease activity for the children with CD or IBD-U favoring CD was scored using the Pediatric Crohn’s Disease Activity Index (PCDAI).^22^ The PCDAI is a validated multi-item instrument that includes clinical history, physical examination, growth parameters, and standard laboratory tests. Scores range from 0 to 100, with clinical remission defined as <10, mild disease 10-≤30, and moderate-to-severe disease ≥31. A reduction of ≥12.5 points from baseline following treatment indicates a clinical response.

### Myeloperoxidase Assay

#### Extraction of MPO

Fecal samples were thawed at room temperature and homogenized. Myeloperoxidase was extracted by collecting 10 mg of stool using a RIDA stool collection tube (R-Biopharm AG) and diluting it into 1 mL of extraction buffer containing 0.2% (w/v) cetrimonium bromide (CTAB; Sigma). As a cationic surfactant, CTAB improves the release of MPO from fecal samples while maintaining the inherent enzymatic properties of MPO.^24^ The recovery and stability of fMPO using extraction buffer containing CTAB have been established previously.^17^

#### Measurement of MPO activity and protein

A standard curve was prepared with purified MPO (Planta Natural Products) and used to determine the activity and protein levels in samples. Fecal MPO in extraction buffer diluted 10-fold (v/v) in assay buffer (1% bovin serum albumin [w/v]/0.025% Tween-20 [v/v] in phosphate-buffered saline) was captured by a mouse monoclonal antibody to human MPO (4A4, BioRad). Enzymatic activity was detected by adding hydrogen peroxide (20 μM; ThermoFisher) to Amplex Red (50 μM; Invitrogen, ThermoFisher) in a 50 mM phosphate buffer, pH 7.4, containing 50 mM NaBr. The plate was subsequently washed to remove peroxidase substrates and products. Myeloperoxidase protein was then probed using rabbit polyclonal anti-myeloperoxidase antibody produced in-house coupled to goat anti-rabbit immunoglobulin-biotin conjugate (ThermoFisher) and detected with an avidin-alkaline phosphatase conjugate and p-nitrophenyl phosphate (Sigma). The concentration of fMPO protein was measured by interpolation using the standard curve of purified human MPO to give a measurement of fMPO protein in ng/mL. The final concentration of fMPO (µg/g) was calculated by accounting for the stool extraction and ELISA plate dilution factors.

#### Measurement of C-reactive protein

C-reactive protein (CRP) was measured by immunoturbidimetric method on a Beckman Coulter AU5822 analyzer using Beckman Coulter reagents in venous blood samples at the Canterbury Health Laboratories as part of routine clinical assessment. An elevated or abnormal CRP level is >5 mg/L.

#### Measurement of fCal

Fecal samples were thawed at room temperature and homogenized prior to calprotectin extraction using the Calpro Easy Extract device and commercial Calpro extraction buffer (Calpro AS). Fecal calprotectin concentration was then measured by commercial enzyme-linked immunosorbent assay (ELISA) as per the manufacturer’s instructions (CALPRO ELISA, Calpro AS).

#### Measurement of glutathione sulfonamide

Glutathione sulfonamide was measured in urine using stable isotope dilution mass spectrometry as outlined previously.^25^ Glutathione sulfonamide concentration was normalized to the specific gravity of the corresponding urine sample as described previously.^9^

### Statistical Analysis

Statistical analyses and graphs from this data were created using GraphPad Prism version 10.0.2 (GraphPad Software Inc.). Correlations between biomarkers and disease activity scores, and between fMPO, fCal, and CRP were performed using Spearman rank correlations. Kruskal–Wallis nonparametric analysis of variance (ANOVA) assessed for differences in fMPO activity and protein and fCal between the 4 groups: healthy siblings, non-IBD, inactive CD, and active CD. Receiver-operator-characteristic curves assessed the precision of biomarkers (fMPO activty, fMPO protein, fCal, and CRP) in predicting IBD and disease activity.

To compare ROC curves and determine whether 1 biomarker was superior, paired data from only the cases with all 4 measures analyzed (MPO activity, MPO protein, calprotectin, CRP) was used to assess paired-sample area difference under the ROC curves. Logistic regression analysis determined whether combining fMPO and CRP was superior to fCal alone at predicting the presence of CD and disease activity.

## Results

### Description of Study Participants

Samples were available from 143 children. Ninety-three children were diagnosed with IBD (88 with CD, and 5 with IBD-U favoring CD) of which 58 were male (62%) and 35 were female (38%). The higher proportion of males in this cohort is a known feature among children diagnosed with IBD. Of those diagnosed, 51 had active disease (40 mild, 10 moderate-to-severe, 1 of unknown severity), and 42 had inactive disease. Fifteen were classified as controls without IBD, and 35 were healthy sibling controls (Table 1).

**Table 1.** Description of demographics, disease activity and biomarker concentrations in study participants.

	Controls	Non-IBD	Inactive CD	Active CD
Total number of participants	35	15	42	51
Mean age in years (SD)	11.7 (3)	12.8 (3.1)	12.3 (3.3)	11.6 (3.8)
Female participants (%)	22 (63)	7 (47)	15 (36)	20 (39)
Median time since diagnosis (years, range)			2.4 (0.1-12.1)	0.2 (0-11.1)
Median PCDAI (IQR)			0 (0-3.75)	21.25 (15-30)
Median fMPO activity (µg/g, IQR)	0 (0-1.2)	0 (0-1.6)	2.1 (0-5.2)	15.4 (4.5-30)
Number analyzed for fMPO activity (%)	34 (97)	13 (87)	41 (98)	48 (94)
Median fMPO protein (µg/g, IQR)	0 (0-0.7)	0 (0-1.3)	2.3 (0-10.3)	18.9 (9.6-52)
Number analyzed for fMPO protein (%)	34 (97)	13 (87)	41 (98)	48 (94)
Median fCal (µg/g, IQR)	39.9 (14.4-80.9)	45.8 (17-75.9)	253.8 (93.2-568)	2851 (1641-4861)
Number analyzed for fCal (%)	20 (57)	12 (80)	30 (71)	36 (71)
Median serum CRP (mg/L, IQR)	3 (3-3)	3 (1.5-3)	3 (3-3)	3.5 (3-23)
Number analyzed for CRP (%)	13 (37)	12 (80)	39 (93)	48 (94)
Median uGSA (µM, IQR)	0.1 (0.08-0.17)	0.15 (0.11-0.3)	0.15 (0.11-0.16)	0.21 (0.15-0.29)
Number analyzed for uGSA (%)	21 (60)	15 (100)	18 (43)	19 (37)

Abbreviations: CRP, C-reactive protein; CD, Crohn’s disease; fCal, fecal calprotectin; fMPO, fecal myeloperoxidase; GSA, glutathione sulfonamide; IBD, inflammatory bowel disease; IQR, interquartile range; PCDAI, Pediatric Crohn’s Disease Activity Index; uGSA, urinary glutathione sulphonamide.

### Correlations Between Biomarkers and With Disease Severity Scores

**Figure 1.**
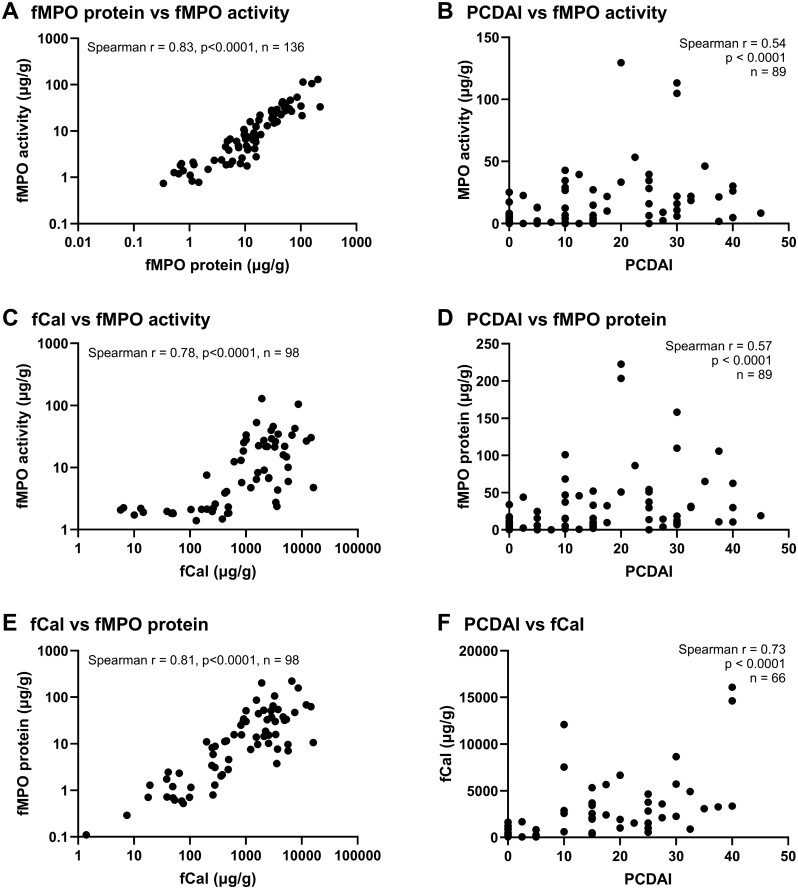
Correlations between biomarkers and disease activity scores. (A) Fecal myeloperoxidase (fMPO) activity correlated with fMPO protein. (B) fMPO activity correlated with Pediatric Crohn’s Disease Activity Index (PCDAI). (C) fMPO activity correlated with fecal calprotectin (fCal). (D) fMPO protein correlated with PCDAI. (E) fMPO protein correlated with fCal. (F) fCal correlated with PCDAI.

Fecal MPO protein concentration was correlated with fMPO activity to determine whether fMPO measured was in the intact active form. In the samples analyzed (*n* = 136) fMPO protein correlated with fMPO activity (*r* = 0.83, *P* < .0001; Figure 1A). The average specific activity of fMPO ([fMPO activity]/[fMPO protein]) was 65% (±29% SD) indicating that the majority of fMPO measured was active. Fecal MPO activity and fMPO protein also correlated with fCal levels (*r* = 0.78, *P* < .0001, and *r* = 0.81, *P* < .0001, respectively; Figure 1C and E). Fecal MPO activity and fMPO protein correlated with PCDAI scores (*r* = 0.54, *P* < .0001, and *r* = 0.57. *P* < .0001, respectively; Figure 1B and D). However, a stronger correlation was observed between fCal and PCDAI scores (*r* = 0.73, *P* < .0001; Figure 1F). CRP levels also correlated with PCDAI scores (*r* = 0.58, *P* < .0001). In the subgroup of children with active CD (PCDAI > 10) fMPO activity, fMPO protein, and fCal did not correlate with PCDAI scores (*r* = 0.22, *P* = .13, *P* = 48; *r* = 0.26, *P* = .07, *n* = 48; and *r* = 0.27; *P* = .11, *n* = 36, respectively). Alternatively, CRP correlated with PCDAI scores in this group (*r* = 0.49, *P* = .0005, *n* = 48). Routinely assessed neutrophil concentrations in the blood were available for a subset of children (*n* = 81) at the time of recruitment. There was no correlation between fMPO activity or fMPO protein with peripheral neutrophil concentration, indicating that although neutrophil concentration is elevated at sites of infection or inflammation it is not necessarily elevated systemically in those with active disease.

### Evaluation of fMPO as a Biomarker of Disease Activity

**Figure 2.**
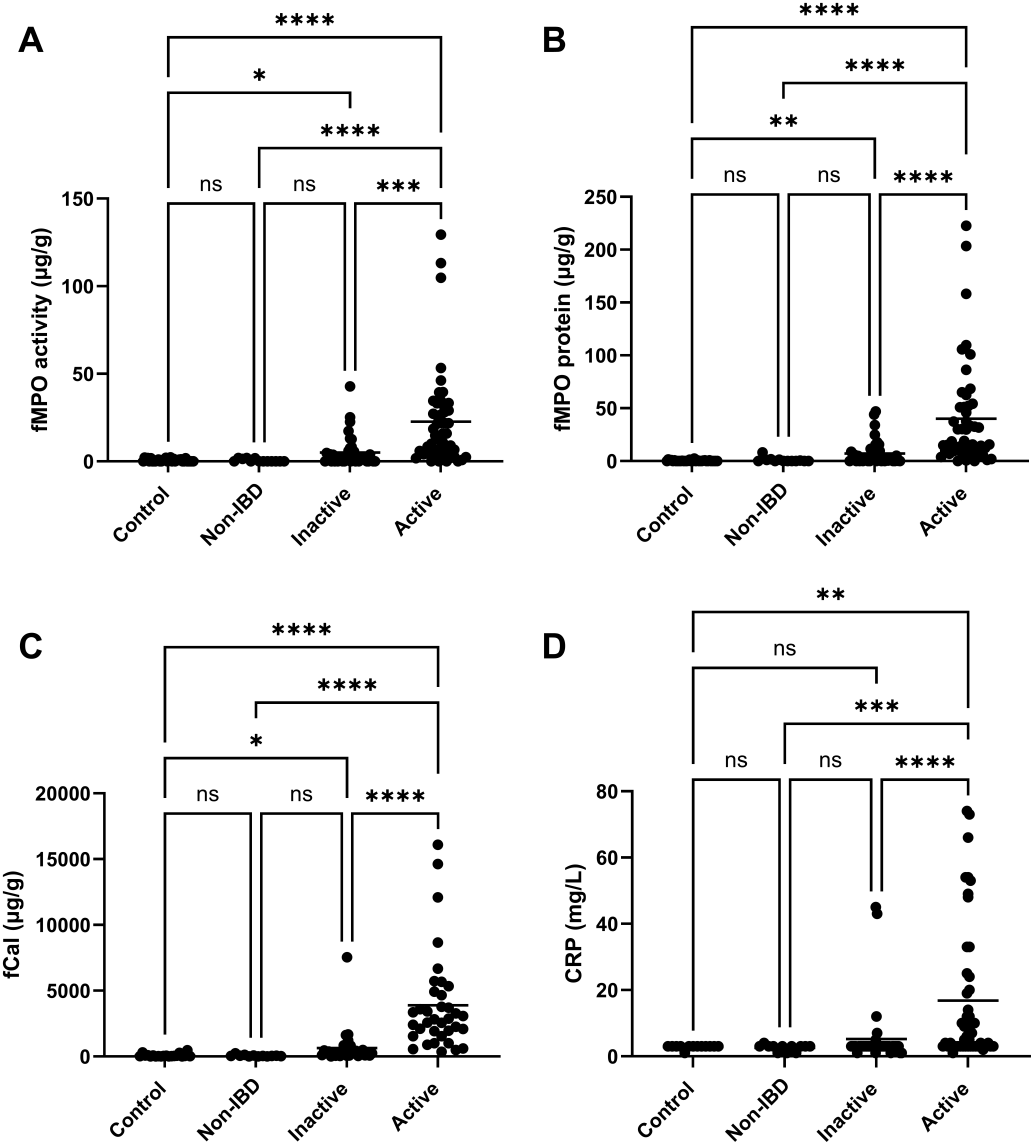
Markers of inflammation in samples from children with and without Crohn’s disease (CD). Samples were grouped into those who were healthy siblings of children with CD (controls), individuals with symptoms suggestive of inflammatory bowel disease (IBD) who underwent investigations and in whom IBD was excluded (non-IBD), and those with inactive or active CD. The concentration of (A) fecal myeloperoxidase (fMPO) activity (control *n* = 34, non-IBD *n* = 13, inactive *n* = 41, active *n* = 48), (B) fMPO protein (control *n* = 34, non-IBD *n* = 13, inactive *n* = 41, active *n* = 48) (C) fecal calprotectin (fCal) (control *n* = 20, non-IBD *n* = 12, inactive *n* = 30, active *n* = 36), and (D) C-reactive protein (CRP) measured in the blood (control *n* = 13, non-IBD *n* = 12, inactive *n* = 39, active *n* = 48) was determined. Each data point represents a different individual and the mean in each group is represented by the line. Differences between groups were determined using Kruskal–Wallis nonparametric analysis of variance (ANOVA). Significant differences between groups represented by: *P < .05, **P < .01, ***P < .001, ****P < .0001.

Fecal MPO activity, fMPO protein, fCal, and CRP levels were all significantly higher in individuals with active disease when compared to healthy sibling controls, the non-IBD group, and those with inactive disease (Figure 2). In addition, fMPO activity, fMPO protein, and fCal levels were significantly lower in healthy sibling controls compared to those with inactive disease (*P* = .004, *P* = .004, and *P* = .02, respectively; Figure 2A-C). The fMPO activity assay was less sensitive than that of the fMPO protein. As a result, fMPO activity levels in several of the control and non-IBD samples were below the limit of detection for this assay and therefore reported as 0, while measurable levels of fMPO protein were detected.

**Figure 3.**
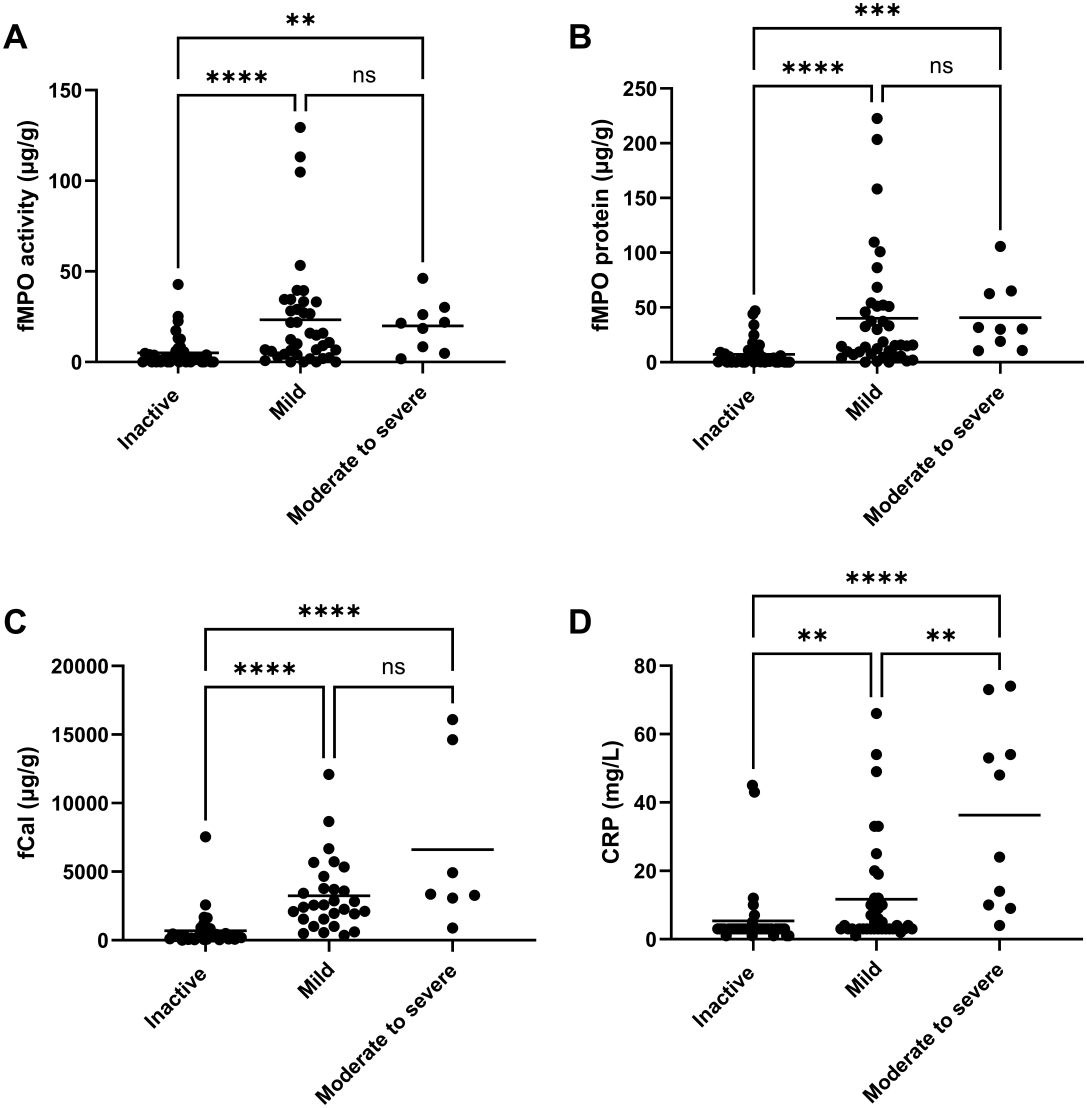
Markers of inflammation in samples from children with inactive or active Crohn’s disease (CD). Samples were grouped into those inactive, mild, or moderate-to-severe disease severity based on their Pediatric Crohn’s Disease Activity Index (PCDAI) score. The concentration of (A) fecal myeloperoxidase (fMPO) activity (inactive *n* = 41, mild *n* = 39, moderate-to-severe *n* = 9), (B) fMPO protein (inactive *n* = 43, mild *n* = 39, moderate-to-severe *n* = 9), (C) fecal calprotectin (fCal) (inactive *n* = 32, mild *n* = 29, moderate-to-severe *n* = 7), and (D) C-reactive protein (CRP) measured in the blood (inactive *n* = 40, mild *n* = 38, moderate-to-severe *n* = 10) was determined. Each data point represents a different individual and the mean in each group is represented by the line. Differences between groups were determined using Kruskal–Wallis nonparametric analysis of variance (ANOVA). Significant differences between groups represented by: *P < .05, **P < .01, ***P < .001, ****P < .0001.

Subgroup analysis separated individuals with CD into those with inactive, mild, or moderate-to-severe disease based on their PCDAI score (Figure 3). A difference in fMPO activity and fMPO protein levels was observed between those with inactive versus mild disease (*P* < .0001, respectively; Figure 3A and B). There was also a difference in fMPO activity and fMPO protein levels between those with inactive versus moderate-to-severe disease (*P* = .003 and *P* = .0003, respectively). There was no significant difference between fMPO activity or fMPO protein levels in those with mild versus moderate-to-severe disease.

When assessing fCal levels in the different disease activity groups, there was a difference between those with inactive versus mild disease (*P* < .0001). There was also a difference in fCal levels between those with inactive versus moderate-to-severe disease (*P* < .0001; Figure 3C). C-reactive protein levels were higher in those with mild or moderate-to-severe disease compared to those with inactive disease (*P* = .0042 and *P* < .0001, respectively; Figure 3D). There was also a difference in the mean CRP levels between those with mild (mean CRP = 11.7 mg/L) versus moderate-to-severe disease (mean CRP = 36.3 mg/L; *P* = .007).

### Receiver-Operative Characteristic Analysis of fMPO Activity, fMPO Protein, fCal, and CRP With Disease Activity

**Figure 4.**
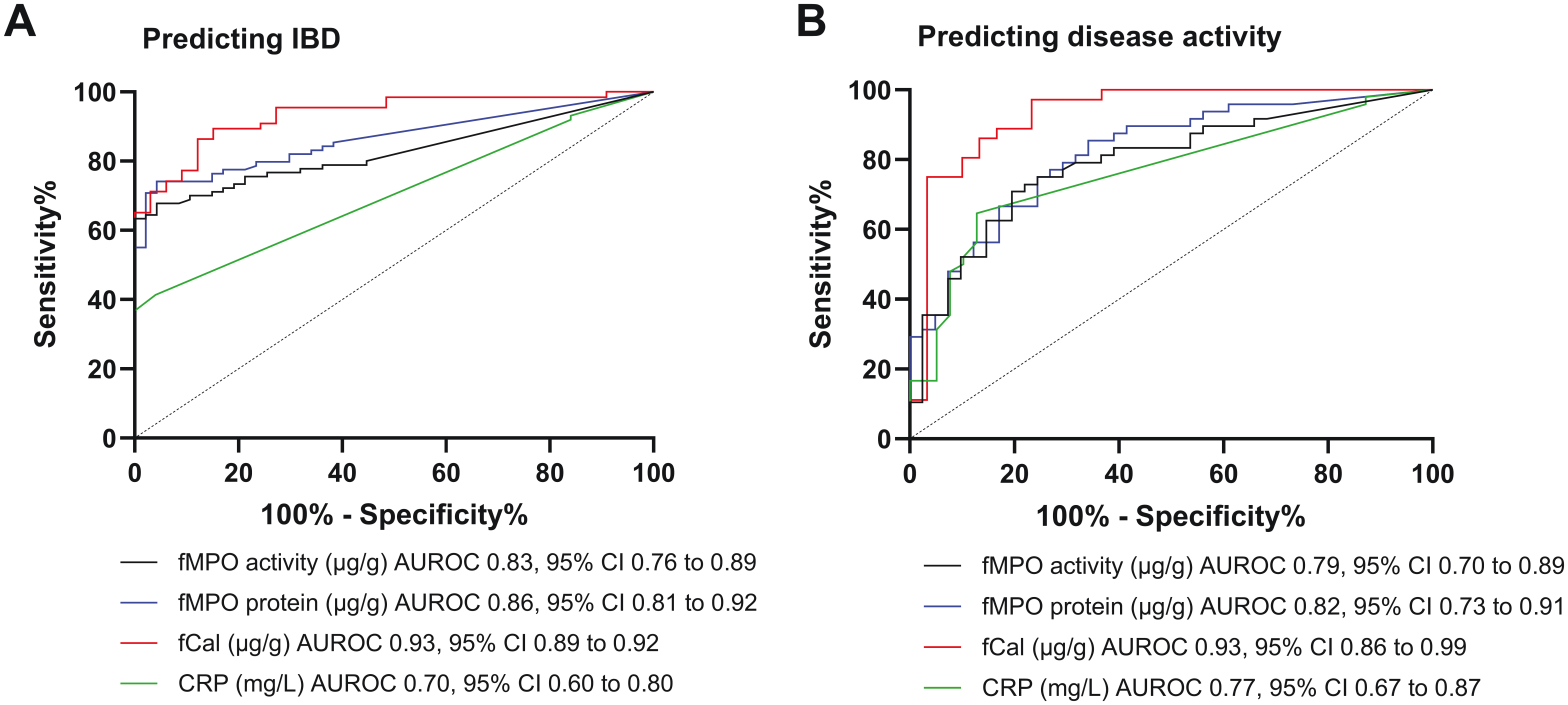
Predicting Crohn’s disease (CD) and CD activity in children using fecal myeloperoxidase (fMPO) activity, fMPO protein, fecal calprotectin (fCal), or C-reactive protein (CRP) biomarkers. Area under the receiver-operating characteristic (AUROC) curve and 95% CI are shown for the receiver-operative characteristic (ROC) analysis for the ability of fMPO activity, fMPO protein, fCal, and CRP to (A) predict the presence or absence of CD and (B) predict disease activity.

Receiver-operative-characteristic analysis demonstrated that fMPO activity, fMPO protein, and fCal were all effective at detecting CD in children (Table 2, Figure 4A). C-reactive protein was not as effective at discriminating between those with and without CD (Table 2, Figure 4A). All the biomarkers analyzed were strong predictors of disease activity in children and discriminated between those with inactive or active disease (Table 2, Figure 4B). Fecal Cal was the most effective predictor of disease activity (area under the receiver-operating characteristic [AUROC] = 0.93, 95% CI, 0.89-0.98, *P* < .0001).

**Table 2.** Area under the AUROC for biomarkers (fMPO activtiy, fMPO protein, fCal, and CRP) to predict the presence of CD and disease activity.

Predicting IBD					
	AUROC	95% CI	*P*-value	Controls (*n*)	CD (*n*)
fMPO activity	0.83	0.76-0.89	**<.0001**	47	90
fMPO protein	0.86	0.81-0.92	**<.0001**	47	89
fCal	0.93	0.89-0.98	**<.0001**	33	66
CRP	0.70	0.60-0.80	**.002**	25	87

Predicting disease activity					
	AUROC	95% CI	*P*-value	Inactive (*n*)	Active (*n*)
fMPO activity	0.79	0.70-0.89	**<.0001**	41	48
fMPO protein	0.82	0.73-0.91	**<.0001**	41	48
fCal	0.93	0.86-1.00	**<.0001**	30	36
CRP	0.77	0.67-0.87	**<.0001**	39	48

Abbreviations: AUROC, area under the receiver-operating characteristic; CRP, C-reactive protein; CD, Crohn’s disease; fCal, fecal calprotectin; fMPO, fecal myeloperoxidase; IBD, inflammatory bowel disease.

When comparing ROC curves from paired data (using only those cases with all 4 biomarkers analyzed), fCal was shown to be superior to fMPO activity and fMPO protein at predicting the presence of CD (fCal AUROC = 0.96, *P* < .0001; fMPO activity AUROC = 0.87, *P* < .0001; fMPO protein AUROC = 0.89, *P* < .0001) or disease activity (fCal AUROC = 0.93, *P* < .0001; fMPO activity AUROC = 0.83, *P* < .0001; fMPO protein AUROC = 0.84, *P* < .0001; Supplementary Table 1). Although the fMPO protein sandwich ELISA was more sensitive than the fMPO activity assay, there was no significant difference between the ability of fMPO activity or fMPO protein to predict the presence of CD or disease activity. Fecal Cal and fMPO were superior to CRP in both instances (Supplementary Table 1). In addition, logistic regression analysis determined that combining the results of fMPO activity, fMPO protein, and CRP was not significantly better than fCal alone at predicting the presence of CD or disease severity (Supplementary Table 2).

### Optimal Cutoff Points of Biomarkers in Assessing Disease Activity

The Youden index (sensitivity + specificity − 1) was used to calculate the optimal cutoff points of fMPO activity, fMPO protein, fCal, and CRP to predict the presence of CD and to predict disease severity (Table 3). An fMPO activity cutoff value of 2.1 µg/g predicted the presence of CD with sensitivity of 68% and specificity of 96%, while a cutoff of 6.3 µg/g predicted disease activity with sensitivity of 71% and specificity of 80%. Similarly, an fMPO protein cutoff value of 1.9 µg/g predicted the presence of CD with sensitivity of 74% and specificity of 96%, while a cutoff of 9.7 µg/g predicted disease activity with sensitivity of 75% and specificity of 76%. In comparison, the fCal cutoff values required to accurately predict the presence of CD and disease severity were much higher than the clinically accepted cutoff value of 50 µg/g, at 145.2 µg/g (86/88% sensitivity and specificity), and 489.7 µg/g (97/77% sensitivity and specificity), respectively. Using the clinically accepted fCal cutoff of 50 µg/g to predict the presence of CD altered the sensitivity/specificity to 95/58% suggesting that a 50 µg/g fCal cutoff would accurately designate an individual who does not have CD only 58% of the time. Similarly, an fCal cutoff of 50 µg/g to predict disease activity had a sensitivity of 100% but the specificity decreased to just 10%, indicating that this cutoff is not able to accurately identify those without active disease. C-reactive protein was the least effective marker at predicting the presence of CD and distinguishing between inactive and active disease, with the optimal cutoff identified as 3.5 mg/L for both with a sensitivity and specificity of 41/96 and 65/87, respectively.

**Table 3.** Optimal cutoff values for predicting CD and disease activity.

Predicting IBD				
	AUROC	95% CI	Optimal cutoff points	Sensitivity/specificity (%)
fMPO activity	0.83	0.76-0.89	2.1 µg/g	68/96
fMPO protein	0.86	0.81-0.92	1.9 µg/g	74/96
fCal	0.93	0.89-0.98	145.2 µg/g	87/88
CRP	0.70	0.60-0.80	3.5 mg/L	41/96

Predicting disease activity				
	AUROC	95% CI	Optimal cutoff points	Sensitivity/specificity (%)
fMPO activity	0.80	0.70-0.89	6.3 µg/g	71/80
fMPO protein	0.82	0.73-0.91	9.7 µg/g	75/76
fCal	0.93	0.86-1.00	489.7 µg/g	97/77
CRP	0.77	0.67-0.87	3.5 mg/L	65/87

Abbreviations: AUROC, area under the receiver-operating characteristic; CRP, C-reactive protein; CD, Crohn’s disease; fCal, fecal calprotectin; fMPO, fecal myeloperoxidase; IBD, inflammatory bowel disease.

### Detection of GSA in Urine as an Indicator of Hypochlorous Acid Production

The oxidative metabolite of glutathione, GSA, was measured in the urine of study participants to confirm that MPO was active and generating hypochlorous acid. Urine samples were available from 37 children with CD (18 inactive) and 36 controls (21 healthy siblings and 15 unrelated well children). Urinary GSA correlated moderately with fMPO activity (*r* = 0.39, *P* = .001) and fMPO protein (*r* = 0.41, *P* = .0005; Supplementary Figure 1A and B). Elevated uGSA levels were detected in those with active disease (mean, range of control = 0.12, 0.05-0.21; non-IBD = 0.19, 0.09-0.39; inactive = 0.15, 0.08-0.24; active = 0.30, 0.11-1.43; Supplementary Figure 1C). Furthermore, analysis of uGSA values across disease activity groups (inactive, mild, or moderate-to-severe PCDAI scores) demonstrated elevated levels in those with mild disease compared to those with inactive disease (mean, range of 0.25, 0.12-0.82, and mean, range of 0.15, 0.08-0.24, respectively, *P* < .05; Supplementary Figure 1D). Similarly, uGSA levels were elevated in those with moderate-to-severe disease compared to those with inactive disease (mean, range of 0.24, 0.11-0.29, and mean, range of 0.15, 0.08-0.24, respectively *P* < .05). However, there was no significant difference between levels of uGSA in those with mild or moderate-to-severe disease (mean, range of 0.25, 0.12-0.82, and mean, range of 0.24, 0.11-0.29, respectively). A larger sample size in the moderate-to-severe group would strengthen the analysis.

## Discussion

The data from the present study show that fMPO is an effective noninvasive marker of inflammation in children with CD. Fecal MPO activity and fMPO protein levels can predict those patients with CD and those who have active disease. Although fCal was significantly better than fMPO activity and fMPO protein at predicting CD and disease severity, a much higher fCal cutoff than the clinically accepted cutoff of >50 µg/g was required to accurately predict the presence of disease and disease activity in this pediatric cohort. The high calprotectin levels detected in some of the healthy children and the previously reported variation in calprotectin levels in younger children highlight the potential superiority of fMPO as a noninvasive marker of inflammation in pediatric patients. Urinary GSA, an oxidative metabolite produced by hypochlorous acid, was significantly correlated with fMPO and was higher in those with active compared to inactive disease, confirming that MPO is predominantly active and generating hypochlorous acid at inflammatory sites.

Strategies for the management of CD are shifting away from simply controlling symptoms toward achieving complete clinical and endoscopic remission, with the aim of preventing disease progression and future complications.^26^ Endoscopy is a critical tool in the evaluation and management of CD. However, this procedure is expensive for the health system and invasive for patients.^27^ New treatment goals aim for tight disease control using therapeutic monitoring and early intervention.^26^ The use of effective noninvasive inflammatory markers for disease monitoring is critical to achieve this treatment goal as the invasiveness of endoscopic examination represents a strong limitation to their frequent use, especially in pediatric patients.

The serum immune marker CRP is commonly used in the evaluation of many inflammatory conditions and is the most widely used serum indicator of inflammation in IBD. It is produced in the liver as an acute phase reactant following interleukin (IL)-6 stimulation.^28^ Comparable to this study, CRP levels have been shown to correlate with disease activity,^29^ however, CRP is not disease specific and can be elevated in many diseases with acute inflammation. Therefore, it must be used in conjunction with relevant clinical information, including additional biomarkers, symptoms, and endoscopy for an accurate diagnosis of IBD or disease activity. In some cases, normal CRP levels have been observed in patients with active disease,^30^ and normal CRP levels have been reported in asymptomatic patients with mild mucosal lesions. Interestingly, 1 study reported that 28% of children with CD and 42% with UC had normal CRP levels at diagnosis.^31^ Results from our study support these findings by demonstrating that CRP was less effective than fMPO and fCal at predicting the presence of CD and disease activity. Consequently, although CRP plays a role in the assessment of disease activity in patients with IBD it has various limitations and must be used in conjunction with additional clinical information for an accurate assessment of the disease state.

Fecal calprotectin has been extensively studied as a noninvasive marker of inflammation in IBD. Numerous studies have demonstrated that it is an effective marker of disease activity and can predict disease relapse and response to treatment.^2,32^ Elevated fCal, especially over 500 µg/g, is suggestive of IBD.^33^ However, when inflammation is mild, the interpretation of the fCal level can be more difficult.^33^ An fCal result of <50 µg/g is generally considered to be in the normal range and the intestine would be considered noninflamed. However, when mild inflammation is predicted within an fCal range of 50-200 µg/g, the clinical situation occurring can be unclear. In this range, fCal levels can vary significantly from day to day,^34^ be increased with the use of nonsteroidal anti-inflammatory drugs,^35^ with enteric infections,^36^ or also in preschool children.^37^ Results from the present study align with these observations, with a cutoff of around 490 µg/g required to discriminate between those with inactive versus active disease. Mild elevation of fCal was also observed in samples from many of the control participants. Recent work has also highlighted that calprotectin is susceptible to oxidation by hypochlorous acid rendering the protein more susceptible to proteolysis.^4,5^ Degradation of calprotectin at sites of infection and inflammation may therefore result in an underestimation of the true level of inflammation furthering the complexity of fCal interpretation. The reported variability of fCal levels in younger children^3,38,39^ and the complexities in the interpretation of fCal in those with levels suggestive of mild disease^33^ highlights the potential limitations of using fCal in the pediatric setting.

Myeloperoxidase has considerable potential as a noninvasive marker of inflammation in IBD due to its high abundance at inflammatory sites. However, there are limited studies assessing the role of MPO as a biomarker in IBD. A recent study in adults with IBD demonstrated that fMPO was an accurate marker of gut inflammation when compared to ileocolonoscopy and performed comparably to fCal.^17^ Fecal MPO levels were able to predict moderate-to-severe disease activity, declined following initiation of biologic therapies, and could predict a more complicated disease course. However, both fCal and fMPO had lower diagnostic accuracy at identifying individuals with CD when compared to UC who had mild endoscopic activity. Although fMPO had lower diagnostic accuracy in adults with CD,^17^ fMPO performed well in this CD pediatric cohort and was able to predict the presence of CD and disease activity. In addition, the measurement of detectable levels of fMPO activity and protein in individuals with inactive disease was of interest and highlights that some may have underlying inflammation but not yet be displaying clinical symptoms. As such, future work exploring whether detectable levels of fMPO in this group are predictive of subsequent colonic flares is warranted. The use of fMPO testing for disease monitoring may inform pre-emptive adjustment of prescribed medications ensuring the maintenance of clinical remission.

Neutrophil infiltration is a histological feature of IBD, with neutrophils recruited and accumulating in the gastrointestinal wall releasing inflammatory cytokines and reactive oxygen species.^8^ Myeloperoxidase may be a key player contributing to the pathogenesis of IBD via its production of the potent oxidant hypochlorous acid. This abundant neutrophil granule enzyme uses hydrogen peroxide to oxidize chloride to hypochlorous acid.^6^ The production of hypochlorous acid plays an important role in the innate immune response, aiding in the killing of phagocytosed pathogens. However, hypochlorous acid can also react indiscriminately with a wide range of biological molecules including proteins, carbohydrates, lipids, and nucleic acids.^7^ The unregulated production of reactive oxygen species such as hypochlorous acid in IBD is thought to result in chronic colitis and adverse alterations in bowel structure and function.^8^ The high abundance of neutrophil-derived cytokines and resulting oxidation products located at inflammatory sites have major potential as accurate biomarkers of inflammation in IBD.

The current study shows that fMPO activity and fMPO protein were significantly correlated, indicating that the majority of MPO is intact and active. This finding was further supported by the detection of urinary GSA, as GSA formation occurs primarily via hypochlorous acid-specific oxidation of glutathione. In addition, urinary GSA was significantly correlated with fMPO activity and fMPO protein and was elevated in those with active compared to inactive disease indicating that GSA is likely formed at inflammatory sites and subsequently excreted in the urine. However, detection of background levels of GSA in urine samples from all participants suggests GSA is formed by other routes besides the generation of hypochlorous acid at sites of inflammation. Nonetheless, the detection of elevated urinary GSA levels in those with active inflammation is an exciting and novel finding supporting the theory that excess MPO-derived hypochlorous acid is present in patients with active IBD.

One limitation of this study was the small number of study participants with moderate-to-severe disease. Additional participants in that disease group are likely to have strengthened the study by further optimizing biomarker cutoff accuracy to correctly distinguish between individuals with mild or moderate-to-severe disease. In addition, external validation of the findings in this study in additional cohorts is essential to optimize biomarker thresholds to accurately predict the presence of disease and disease severity. Disease location can also have an impact on the ability of biomarkers to accurately reflect disease severity, with previous studies indicating higher neutrophil activity in colonic than in ileal CD.^40^ Future work in a larger pediatric cohort assessing the effect of disease location on fMPO levels would be vital to further validate the use of this biomarker in clinical practice.

The collection and handling of fecal samples were carried out to reflect real-world clinical practice. After collection, participants were provided with clear instructions to temporarily store samples in an insulated cool pack in the refrigerator or freezer prior to delivery to a local laboratory and storage at −80 °C. Incorrect storage of fecal samples prior to delivery to a laboratory may result in inaccurate MPO protein and activity results. Previous validation experiments indicated that fMPO was stable at 4 °C for 7 days; however, a significant decrease in fMPO protein concentration was observed when samples were stored for 3 or more days at 25 °C.^17^ In addition, fMPO activity was thought to be even more sensitive to temperature changes. Consequently, the variation in fMPO-specific activity observed in the current cohort may be attributed to variations in sample storage prior to delivery to a laboratory. It may be possible to circumvent some of these limitations through the use of rapid methods to detect fecal markers at home or in the clinic. Rapid testing for fCal is becoming more readily available, and based on the findings of this study the exploration and development of similar methods to rapidly measure fMPO as an alternative is warranted.

In conclusion, the current study has demonstrated that fMPO activity and protein are effective markers of disease activity in children with CD. A much higher fCal cutoff than the clinically accepted cutoff of >50 µg/g was required to accurately predict the presence of CD and disease activity in this pediatric cohort. Fecal MPO may be preferable to calprotectin due to the affordability of this assay and rapid turnaround time for results, particularly for fMPO activity. Detection of glutathione sulfonamide in urine and its correlation with fMPO and elevation in those with active CD suggests that MPO is active in children with current disease exacerbation and generates hypochlorous acid at inflammatory sites. Although fMPO shows promise as a biomarker of inflammation in children with CD, further research is needed to validate its use as a diagnostic and prognostic marker of CD in clinical practice.

## Supplementary Data

Supplementary data is available at *Inflammatory Bowel Diseases* online.

izae262_suppl_Supplementary_Material

## Data Availability

Data are available upon request to the corresponding author.

## Abbreviations:

ANOVA: analysis of variance
AUROC: area under the receiver-operating characteristic
CTAB: cetrimonium bromide
CRP: C-reactive protein
CD: Crohn’s disease
CF: cystic fibrosis
ELISA: enzyme-linked immunosorbent assay
fCal: fecal calprotectin
fMPO: fecal myeloperoxidase
GSA: glutathione sulfonamide
IBD: inflammatory bowel disease
IBD-U: inflammatory bowel disease unclassified
IL-6: interleukin-6
MPO: myeloperoxidase
GSSG: oxidized glutathione
PCDAI: Pediatric Crohn’s Disease Activity Index
ROC: receiver-operator characteristic
REDCap: research electronic data capture
UC: ulcerative colitis
uGSA: urinary glutathione sulfonamide
