# Supplementary material for: Fecal Myeloperoxidase Levels Reflect Disease Activity in Children With Crohn’s Disease
Source: Inflamm Bowel Dis. 2024 Nov 11;31(3):800–11. doi: 10.1093/ibd/izae262 (PMC11879223; doi:10.1093/ibd/izae262)
Supplement: izae262_suppl_Supplementary_Material [file izae262_suppl_supplementary_material.docx]

**Supplementary Material**

**Supplementary Table 1.** Comparing the accuracy of biomarkers to predict the presence of CD and disease activity.

| **Predicting IBD** | | | | | | | | |
| --- | --- | --- | --- | --- | --- | --- | --- | --- |
| **Test Result Variables** |  |  |  | |  | |  | |
|  | **AUROC** | **p-value** | | **95% CI** | | **Controls (n)** | | **CD (n)** |
| fMPO activity | 0.874 | **<0.0001** | | 0.80 – 0.95 | | 12 | | 62 |
| fMPO protein | 0.886 | **<0.0001** | | 0.81 – 0.96 | | 12 | | 62 |
| fCal | 0.958 | **<0.0001** | | 0.92 – 1.00 | | 12 | | 62 |
| CRP | 0.714 | **0.02** | | 0.58 – 0.85 | | 12 | | 62 |
| **Test Result Pairs** |  |  | |  | |  | |  |
|  | **AUROC Difference** | **p-value** | | **95% CI** | | **Controls (n)** | | **CD (n)** |
| fMPO activity vs fMPO protein | -0.012 | 0.764 | | -0.09 – 0.07 | | 12 | | 62 |
| fMPO activity vs fCal | -0.085 | **0.010** | | -0.15 – -0.02 | | 12 | | 62 |
| fMPO activity vs CRP | 0.160 | **0.011** | | 0.04 – 0.28 | | 12 | | 62 |
| fMPO protein vs fCal | -0.073 | 0.054 | | -0.15 – 0.001 | | 12 | | 62 |
| fMPO protein vs CRP | 0.172 | **0.007** | | 0.05 – 0.30 | | 12 | | 62 |
| fCal vs CRP | 0.245 | **<0.0001** | | 0.12 – 0.37 | | 12 | | 62 |
| **Predicting disease severity** | | | | | | | | |
| **Test Result Variables** |  |  | |  | |  | |  |
|  | **AUROC** | **p-value** | | **95% CI** | | **Inactive (n)** | | **Active (n)** |
| fMPO activity | 0.830 | **<0.0001** | | 0.72 – 0.94 | | 28 | | 34 |
| fMPO protein | 0.838 | **<0.0001** | | 0.74 – 0.94 | | 28 | | 34 |
| fCal | 0.932 | **<0.0001** | | 0.86 – 1.00 | | 28 | | 34 |
| CRP | 0.785 | **<0.0001** | | 0.67 – 0.90 | | 28 | | 34 |
| **Test Result Pairs** |  |  | |  | |  | |  |
|  | **AUROC Difference** | **p-value** | | **95% CI** | | **Inactive (n)** | | **Active (n)** |
| fMPO activity vs fMPO protein | -0.008 | 0.705 | | -0.05 – 0.03 | | 28 | | 34 |
| fMPO activity vs fCal | -0.102 | **0.004** | | -0.17 – -0.03 | | 28 | | 34 |
| fMPO activity vs CRP | 0.045 | 0.434 | | -0.07 – 0.16 | | 28 | | 34 |
| fMPO protein vs fCal | -0.094 | **0.020** | | -0.17 – -0.02 | | 28 | | 34 |
| fMPO protein vs CRP | 0.053 | 0.356 | | -0.06 – 0.17 | | 28 | | 34 |
| fCal vs CRP | 0.147 | **0.011** | | 0.03 – 0.26 | | 28 | | 34 |

**Supplementary Table 2.** Combining biomarkers to predict the presence of CD and disease activity.

| **Predicting IBD** | | | | | | | | |
| --- | --- | --- | --- | --- | --- | --- | --- | --- |
| **Test Result Variables** |  |  |  | |  | |  | |
|  | **AUROC** | **p-value** | | **95% CI** | | **Controls (n)** | | **CD (n)** |
| fMPOa + fMPOp + CRP | 0.913 | **<0.0001** | | 0.85 – 0.98 | | 12 | | 62 |
| fCal | 0.958 | **<0.0001** | | 0.92 – 1.00 | | 12 | | 62 |
| **Test Result Pairs** |  |  | |  | |  | |  |
|  | **AUROC Difference** | **p-value** | | **95% CI** | | **Controls (n)** | | **CD (n)** |
| fMPOa + fMPOp + CRP vs fCal | -0.046 | 0.183 | | -0.11 – 0.02 | | 12 | | 62 |
| **Predicting disease severity** | | | | | | | | |
| **Test Result Variables** |  |  | |  | |  | |  |
|  | **AUROC** | **p-value** | | **95% CI** | | **Inactive (n)** | | **Active (n)** |
| fMPOa + fMPOp + CRP | 0.866 | **<0.0001** | | 0.78 – 0.96 | | 28 | | 34 |
| fCal | 0.93 | **<0.0001** | | 0.86 – 1.00 | | 28 | | 34 |
| **Test Result Pairs** |  |  | |  | |  | |  |
|  | **AUROC Difference** | **p-value** | | **95% CI** | | **Inactive (n)** | | **Active (n)** |
| fMPOa + fMPOp + CRP vs fCal | -0.066 | 0.087 | | -0.14 – 0.01 | | 28 | | 34 |


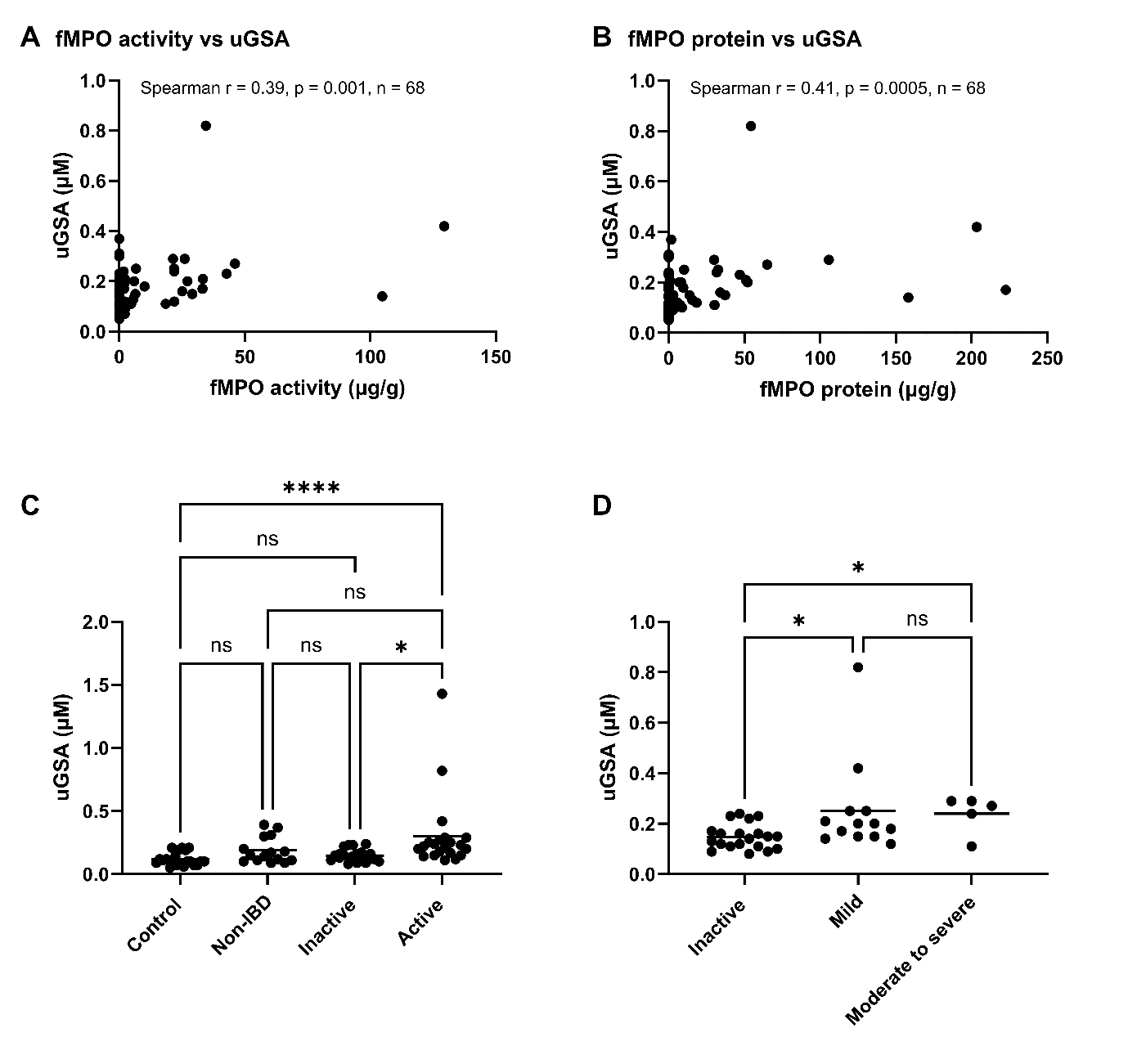


**Supplementary Figure 1. Urinary GSA as a marker of inflammation in children with CD.** Urinary GSA (uGSA) correlated with (A) fMPO activity and (B) fMPO protein. Concentration of uGSA in (C) healthy siblings of children with IBD (controls) (n=21), individuals with symptoms suggestive of IBD who underwent investigations and in whom IBD was excluded (non-IBD) (n=15), and those with inactive CD (n=18) or active CD (n=19), and (D) those with inactive (n=20), mild (n=13), or moderate-to-severe (n=5) disease severity based on their PCDAI score. Each data point represents a different individual and the mean in each group is represented by the line. Differences between groups were determined using Kruskal-Wallis non-parametric ANOVA. Significant differences between groups represented by: *p <0.05, **p <0.01, ***p <0.001, ****p <0.0001.
